# Construction and validation of nomograms for predicting the prognosis of grade 3 endometrial endometrioid adenocarcinoma cancers: a SEER-based study

**DOI:** 10.1080/21655979.2021.1922247

**Published:** 2021-05-11

**Authors:** Xiaofei Liu, Junbo Zhao, Zhiwei Sun, Guangwei Wang

**Affiliations:** a Department of Obstetrics and Gynecology, Shenyang Women’s and Children’s Hospital, Shenyang, China; b Department of Obstetrics and Gynecology, Shengjing Hospital of China Medical University, Shenyang, China

**Keywords:** EAC, prognosis, SEER, nomogram

## Abstract

Most cases of endometrial adenocarcinoma (EAC) are diagnosed early and have a good prognosis; however, grade 3 (G3) EACs have poor outcomes. We retrospectively analyzed the data of 11,519 patients with G3 EACs registered between 2004 and 2015 in the Surveillance, Epidemiology, and End Results Program database and constructed a nomogram to guide clinicians in decision-making and accurate prediction of the prognosis. The caret package was used to divide samples into a training set and a validation set. Univariate and multivariate Cox regression analyses were performed, and a nomogram was constructed. A calibration curve was plotted, and a decision curve analysis was performed to verify the accuracy and clinical utility in both cohorts. The Cox regression analysis revealed that age, race, tumor size, number of lymph nodes resected, International Federation of Gynecology and Obstetrics stage, tumor/node stage, and adjuvant therapy were the prognostic factors for G3 EAC, and these were included in the nomogram. The area under the curve values of the training cohort for 1-, 3-, and 5-year were 0.832, 0.798, and 0.784, respectively for the overall survival (OS) group, and 0.858, 0.812, and 0.799, respectively for the cancer specific survival (CSS) group. A nomogram was constructed to predict the survival rate of patients with G3 EACs more accurately. The predictive nomogram will help clinicians manage patients with G3 EACs more effectively in terms of clinical prognosis.

## Graphical abstract

**Figure:**
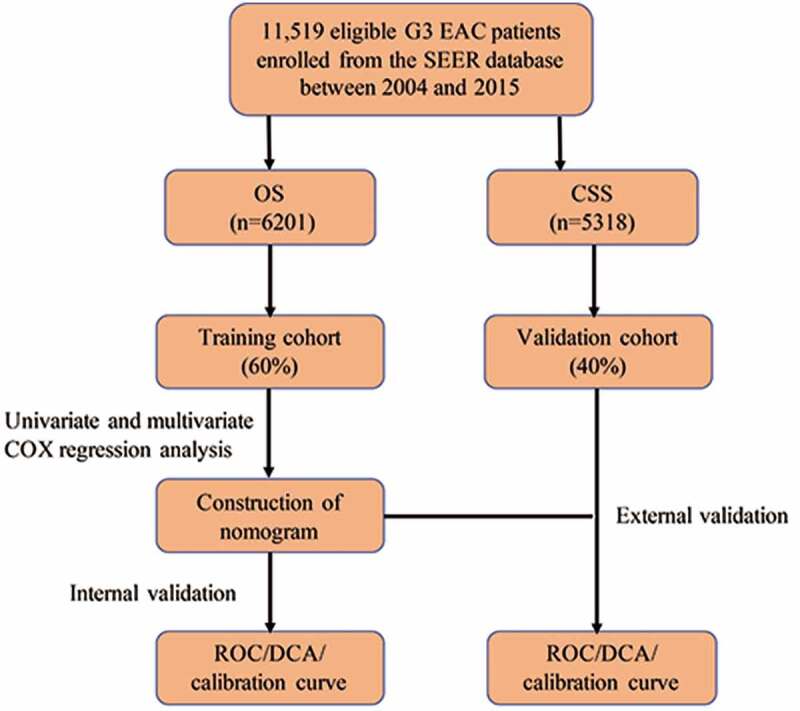

## Introduction

Endometrial carcinomas (ECs) are some of the most common malignant tumors of the female reproductive system. ECs had an estimated incidence of 65,620 new cases and 12,590 deaths in 2020 in the United States [1]. The main clinical presentation of early EC is abnormal vaginal bleeding. While patients with ECs that are diagnosed early have good prognoses, with a 5-year overall survival (OS) of about 90%, the prognoses of patients with advanced or high-grade ECs remain poor [2]. High-grade ECs account for 10–20% of all ECs and 40% of all mortality due to ECs. The pathological types of high-grade EC mainly include grade 3 (G3) endometrial adenocarcinoma (EAC), dedifferentiated carcinoma, undifferentiated carcinoma, clear cell carcinoma, serous carcinoma, mixed adenocarcinoma, and carcinosarcoma [3]. The factors suggested by the International Federation of Gynecology and Obstetrics (FIGO) staging system are currently followed to determine the prognosis of patients with ECs; however, their performance in predicting the individual survival risk is poor due to the low accuracy and omission of independent risk factors, such as age, for the patients’ survival outcomes [4–6]. Therefore, an individualized clinical prediction model for patients with G3 EACs is necessary.

In this study, the clinical characteristics and prognostic factors of patients with G3 EACs were obtained from the Surveillance, Epidemiology, and End Results (SEER) Program database from 2004 to 2015. We aimed to construct G3 EAC nomogram models based on the SEER data and predict the survival to meet the current clinical requirements. Our hypothesis is that a predictive nomogram will help clinicians manage patients with G3 EACs more effectively in terms of accurate clinical prognosis, which is very important to guide decision-making and predicting the prognoses of these patients.

## Materials and methods

### Data sources

Data were downloaded from the SEER Program database, and the patients were from the SEER population-based cancer registry (2004–2015 data set). The demographic and clinical characteristics of the patients were defined as follows: year of diagnosis (2004–2007, 2008–2011, or 2012–2015), race (white, black, or other), marital status (married, unmarried, or other), number of lymph nodes resected (1–10, >10, or none), FIGO stage (I, II, III, or IV), primary tumor stage (T1, T2, T3, or T4), regional lymph node stage (N0 or N1), distant metastases (M0 or M1), and neoadjuvant therapy (chemotherapy alone, radiotherapy alone, combination therapy, or neither therapy).

### Inclusion and exclusion criteria

EAC samples were included according to the following criteria: (1) site recode International Classification of Diseases for Oncology, 3rd edition/WHO 2008 (C540-C543, C548-C549); (2) history code (8380–8383); (3) behavior code (3); (4) G3 (grade distinction); and (5) survival time >30 days. Samples were excluded if the following criteria were noted: (1) missing values and unknown clinical/tissue characteristics; and (2) ECs occurring in addition to other primary malignant tumors.

### Statistical analysis

R 3.6.1 was used for statistical analysis. X-tile software is a new bioinformatics tool suitable for biomarker evaluation and result-based cut point optimization(Yale University, New Haven, USA). That is, different values are used as cutoff values to group for statistical testing. The result with the smallest p-value of the test result can be considered as the best cutoff value. Based on the results of X-tile software, we found that in OS group, when age was classified into 3 subgroups: <64 years, 64–77 years, and >77 years, the prognosis is the most significant among different subgroups (Supplementary Figure 1 abc). When Tumor size was classified as <48 mm, and ≥ 48 mm (Supplementary Figure 1 def), there is a significant prognostic difference between the two groups. Similarly. In CSS group, the best cutoff value for age is: <59 years, 59–69 years, and >69 years (Supplementary Figure1 ghi). The best cutoff value for Tumor size is: <60 mm, and ≥ 60 mm (Supplementary Figure1 jkl). Univariate Cox regression was used to determine the risk factors related to OS and CSS. Patients with a P-value <0.05 in the univariate Cox regression analysis were included in the multivariate Cox regression analysis (variable screening method: bidirectional) to determine the independent prognostic factors and establish nomograms for predicting OS or CSS based on independent factors. Univariate and multivariate analyses were performed using the survival package, receiver operating characteristic (ROC) curves were plotted with the survival ROC package. Nomogram was drawn based on the nomogram function in the rms package.

## Results

### Flowchart of the analysis

**Figure 1.**
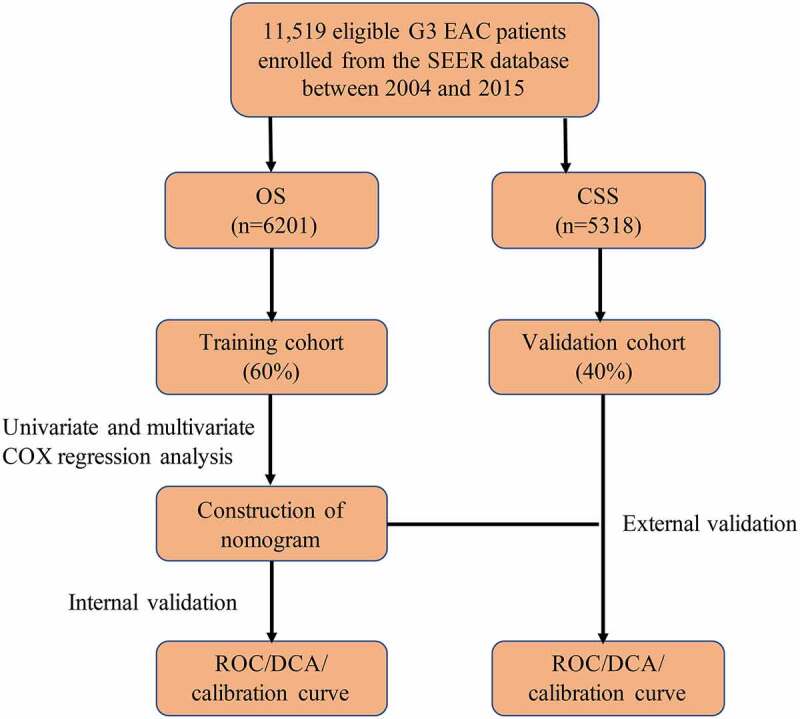
Flow chart of analysis

In order to facilitate the understanding of the research, a methodology flowchart is provided in Figure 1. A total of 11,519 eligible patients with G3 EACs between 2004 and 2015 were enrolled from the SEER Program database. Patients were divided into a training set (OS: n = 3721; CSS: n = 3192) and a validation set (OS: n = 2480; CSS: n = 2126). There were no statistically significant differences in demographic or clinical characteristics between the two groups. Tables 1 and 2 list the demographic data and tumor characteristics of the patients in the OS and CSS groups, respectively. Subsequently, univariate and multivariate Cox regression analyses were performed to construct a nomogram and predict the 1-, 3-, and 5-year survival of the patients. The calibration plot and decision curve analysis (DCA) curve were used to evaluate the accuracy and clinical applicability of the model.

**Table 1.** Clinical information of OS cohort

	Test Cohort	Train Cohort	*p*	X^2^
n	2480	3721		
Age (%)				
<64	1221 (49.23)	1860 (50.0)	0.838	0.35436
64–77	891 (35.93)	1313 (35.3)		
>77	368 (14.84)	548 (14.7)		
Year_of_diagnosis (%)				
2004–2007	728 (29.4)	1068 (28.7)	0.701	0.71169
2008–2011	873 (35.2)	1296 (34.8)		
2012–2015	879 (35.4)	1357 (36.5)		
Race (%)				
White	1974 (79.6)	2954 (79.4)	0.757	0.55656
Black	235 (9.5)	372 (10.0)		
Others	271 (10.9)	395 (10.6)		
Marital_status (%)				
Married	1271 (51.2)	1910 (51.3)	0.965	0.071615
Unmarried	471 (19.0)	697 (18.7)		
Others	738 (29.8)	1114 (29.9)		
Tumor_size (%)				
<4.8 cm	1453 (58.6)	2124 (57.1)	0.25	1.324
≥4.8 cm	1027 (41.4)	1597 (42.9)		
Lymph_nodes_resected (%)				
1–10 nodes	705 (28.4)	1048 (28.2)	0.515	1.3272
>10 nodes	1363 (55.0)	2013 (54.1)		
No	412 (16.6)	660 (17.7)		
FIGO_stage (%)				
Stage I	1354 (54.6)	2090 (56.2)	0.382	3.0644
Stage II	263 (10.6)	359 (9.6)		
Stage III	642 (25.9)	923 (24.8)		
Stage IV	221 (8.9)	349 (9.4)		
T_stage (%)				
T1	1536 (61.9)	2317 (62.3)	0.756	1.1856
T2	354 (14.3)	502 (13.5)		
T3	533 (21.5)	806 (21.7)		
T4	57 (2.3)	96 (2.6)		
N_stage (%)				
N0	2006 (80.9)	3035 (81.6)	0.524	0.4051
N1	474 (19.1)	686 (18.4)		
M_stage (%)				
M0	2286 (92.2)	3421 (91.9)	0.769	0.086276
M1	194 (7.8)	300 (8.1)		
Adjuvant_therapy (%)				
Neither	905 (36.5)	1356 (36.4)	0.189	4.7718
Chemotherapy alone	319 (12.9)	514 (13.8)		
Radiotherapy alone	836 (33.7)	1291 (34.7)		
Combination	420 (16.9)	560 (15.0)

**Table 2.** Clinical information of CSS cohort

	Test Cohort	Train Cohort	p	X2
n	2126	3192		
Age (%)				
<59	742 (34.9)	1079 (33.8)	0.542	1.2247
59–69	735 (34.6)	1149 (36.0)		
>69	649 (30.5)	964 (30.2)		
Year_of_diagnosis (%)				
2004–2007	619 (29.1)	930 (29.1)	0.999	0.002415
2008–2011	749 (35.2)	1126 (35.3)		
2012–2015	758 (35.7)	1136 (35.6)		
Race (%)				
White	1679 (79.0)	2508 (78.6)	0.313	2.3227
Black	228 (10.7)	318 (10.0)		
Others	219 (10.3)	366 (11.5)		
Marital_status (%)				
Married	1092 (51.4)	1630 (51.1)	0.716	0.66823
Unmarried	403 (19.0)	633 (19.8)		
Others	631 (29.7)	929 (29.1)		
Tumor_size (%)				
<6 cm	1490 (70.1)	2251 (70.5)	0.757	0.096026
≥6 cm	636 (29.9)	941 (29.5)		
Lymph_nodes_resected (%)				
1–10 nodes	616 (29.0)	889 (27.9)	0.195	3.2677
>10 nodes	1180 (55.5)	1749 (54.8)		
No	330 (15.5)	554 (17.4)		
FIGO_stage (%)				
Stage I	1207 (56.8)	1724 (54.0)	0.097	6.3224
Stage II	203 (9.5)	325 (10.2)		
Stage III	536 (25.2)	818 (25.6)		
Stage IV	180 (8.5)	325 (10.2)		
T_stage (%)				
T1	1348 (63.4)	1948 (61.0)	0.173	4.9788
T2	290 (13.6)	437 (13.7)		
T3	441 (20.7)	715 (22.4)		
T4	47 (2.2)	92 (2.9)		
N_stage (%)				
N0	1727 (81.2)	2561 (80.2)	0.385	0.75517
N1	399 (18.8)	631 (19.8)		
M_stage (%)				
M0	1968 (92.6)	2915 (91.3)	0.116	2.4751
M1	158 (7.4)	277 (8.7)		
Adjuvant_therapy (%)				
Neither	762 (35.8)	1139 (35.7)	0.224	4.3765
Chemotherapy alone	295 (13.9)	414 (13.0)		
Radiotherapy alone	753 (35.4)	1100 (34.5)		
Combination	316 (14.9)	539 (16.9)

### Independent prognostic factors for G3 EAC

In the OS training set, univariate and multivariate Cox regression analysis of all the variables revealed that age, race, tumor size, number of lymph nodes resected, FIGO stage, tumor/node (T/N) stage, and adjuvant therapy were independent prognostic factors (*P < 0.05*, Table 3). In the CSS training set, univariate and multivariate Cox analyses were also performed for all variables (*P < 0.05*, Table 4). The results showed that age, race, tumor size, number of lymph nodes resected, FIGO stage, T/N stage, and adjuvant therapy were independent prognostic factors of CSS. The results were consistent with those of OS. To visualize these trends, we created a forest plot of the OS (Supplementary Figure 2) and CSS groups (Supplementary Figure 3).

**Table 3.** Univariate and multivariate cox analysis in OS

Variable	Overall Survival(OS)			
	Univariate		Multivariate	
	HR (95% CI)	*p* value	HR (95% CI)	*p* value
Age				
<64	1		1	
64–77	1.77(1.56–2.01)	<0.001	1.78(1.56–2.03)	<0.001
>77	3.22(2.96–3.89)	<0.001	3.05(2.61–3.57)	<0.001
Year of diagnosis				
2004–2007	1		1	
2008–2011	0.89(0.78–1.02)	0.97		
2012–2015	1.08(0.91–1.22)	0.47		
Race				
White	1			
Black	1.50(1.28–1.77)	<0.001	1.29(1.10–1.53)	<0.002
Others	0.83(0.69–1.01)	0.068	0.98(0.80–1.19)	0.81
Marital status				
Married	1			
Unmarried	1.17(1.00–1.36)	0.049	1.07(0.91–1.25)	0.427
Others	1.70(1.51–1.92)	<0.001	1.22(1.07–1.38)	0.003
Tumor size				
<4.8 cm	1			
≥4.8 cm	1.84(1.65–2.05)	<0.001	1.32(1.18–1.48)	<0.001
Lymph nodes resected				
1–10 nodes	1			
>10 nodes	0.68(0.60–0.77)	<0.001	0.78(0.69–0.89)	0.003
No	1.59(1.37–1.84)	<0.001	1.50(1.29–1.75)	<0.001
FIGO stage				
Stage I	1			
Stage II	1.93(1.60–2.33)	<0.001	1.74(1.20–2.51)	0.003
Stage III	2.54(2.23–2.90)	<0.001	1.40(1.06–1.85)	0.02
Stage IV	7.10(6.10–8.27)	<0.001	1.93(1.13–3.30)	0.015
T stage				
T1	1			
T2	1.93(1.65–2.27)	<0.001	1.08(0.79–1.48)	0.630
T3	3.27(2.89–3.70)	<0.001	1.92(1.50–2.46)	<0.001
T4	7.68(6.07–9.72)	<0.001	2.18(1.48–3.22)	<0.001
N stage				
N0	1			
N1	2.37(2.10–2.67)	<0.001	1.85(1.55–2.21)	<0.001
M stage				
M0	1			
M1	4.77(4.12–5.51)	<0.001	1.49(0.95–2.33)	0.08
Adjuvant therapy				
Neither	1		1	
Chemotherapy alone	1.86(1.60–2.17)	<0.001	0.86(0.72–1.02)	0.09
Radiotherapy alone	0.79(0.69–0.90)	<0.001	0.85(0.74–0.98)	0.02
Combination	0.96(0.81–1.14)	0.64	0.64(0.53–0.77)	<0.001

**Table 4.** Univariate and multivariate cox analysis in CSS

Variable	CSS			
	Univariate		Multivariate	
	HR (95% CI)	*p* value	HR (95% CI)	*p* value
Age				
<59	1		1	
59–69	1.33(1.11–1.59)	0.002	1.45(1.21–1.75)	<0.001
>69	1.85(1.55–2.21)	<0.001	2.00(1.64–2.43)	<0.001
Year of diagnosis				
2004–2007	1			
2008–2011	0.95(0.81–1.13)	0.58		
2012–2015	1.08(0.89–1.30)	0.44		
Race				
White	1			
Black	1.65(1.35–2.02)	<0.001	1.33(1.08–1.65)	0.007
Others	0.86(0.68–1.10)	0.24	1.00(0.78–1.28)	0.998
Marital status				
Married	1			
Unmarried	1.27(1.05–1.53)	0.014	0.97(0.80–1.18)	0.78
Others	1.46(1.24–1.71)	<0.001	1.13(0.95–1.34)	0.15
Tumor size				
<6 cm	1			
≥6 cm	2.47(2.15–2.85)	<0.001	1.47(1.26–1.71)	<0.001
Lymph nodes resected				
1–10 nodes	1			
>10 nodes	0.73(0.61–0.86)	<0.001	0.89(0.75–1.06)	<0.001
No	1.78(1.47–2.14)	<0.001	1.87(1.27–1.89)	<0.001
FIGO stage				
Stage I	1			
Stage II	2.08(1.58–2.74)	<0.001	2.24(1.41–3.58)	<0.001
Stage III	4.04(3.37–4.84)	<0.001	2.44(1.77–3.35)	<0.001
Stage IV	12.26(10.09–14.90)	<0.001	3.78(2.04–6.98)	<0.001
T stage				
T1	1			
T2	1.93(1.55–2.41)	<0.001	0.89(0.61–1.29)	0.54
T3	4.49(3.83–5.27)	<0.001	1.47(1.14–1.90)	0.003
T4	9.27(7.02–12.24)	<0.001	1.57(1.03–2.38)	0.036
N stage				
N0	1			
N1	3.22(2.79–3.73)	<0.001	1.86(1.52–2.27)	<0.001
M stage				
M0	1			
M1	6.94(5.88–8.18)	<0.001	1.56(0.93–2.62)	0.09
Adjuvant therapy				
Neither	1			
Chemotherapy alone	2.85(2.35–3.45)	<0.001	0.85(0.72–1.02)	0.16
Radiotherapy alone	0.86(0.72–1.04)	0.12	0.88(0.74–0.98)	0.21
Combination	1.42(1.16–1.75)	<0.001	0.73(0.58–0.91)	0.005

**Figure 2.**
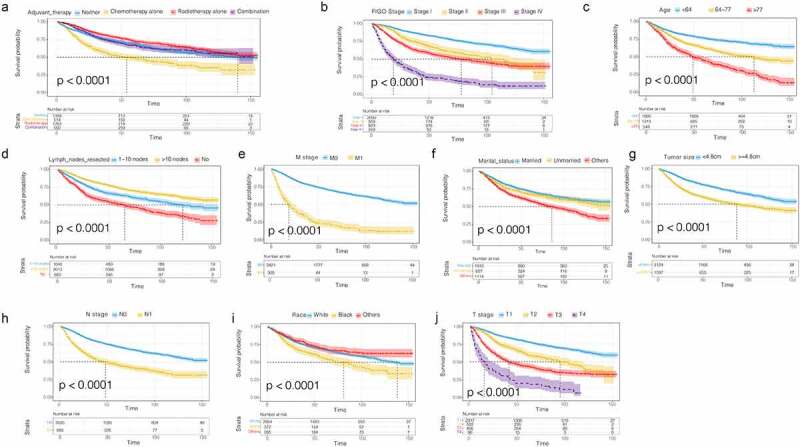
OS survival curves in patients with G3 EACs

**Figure 3.**
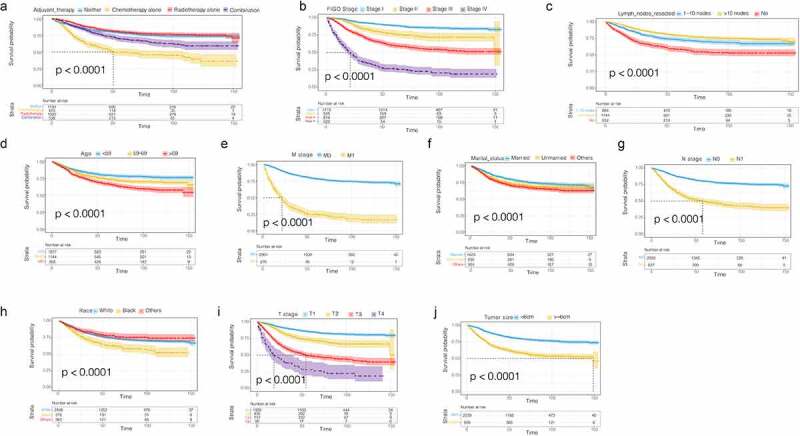
CSS survival curves in patients with G3 EACs

In addition, we plotted the survival curve of each significant variable in the univariate Cox regression analysis of the OS data set. We found that all the variables were related to prognosis except the age at diagnosis (Figure 2). The survival rate of patients treated with postoperative radiotherapy alone was significantly better than those treated with postoperative chemotherapy alone, and the survival benefit of patients treated with combined postoperative chemoradiotherapy was better than that of patients treated with postoperative chemotherapy (Figure 2(a)). The survival rate of the patients was not only affected by age, race, tumor size, number of lymph nodes resected, FIGO stage, and T/N stage (Figure 2(b–e,g,i,j)), but also by marital status. The survival rate of married patients was better than that of unmarried patients (Figure 2(f)). The prognoses of black patients with G3 EACs were generally worse than those of white patients (Figure 2(h)). Consistent results were observed in the CSS univariate survival curve (Figure 3).

### Construction of nomograms related to OS and CSS

**Figure 4.**
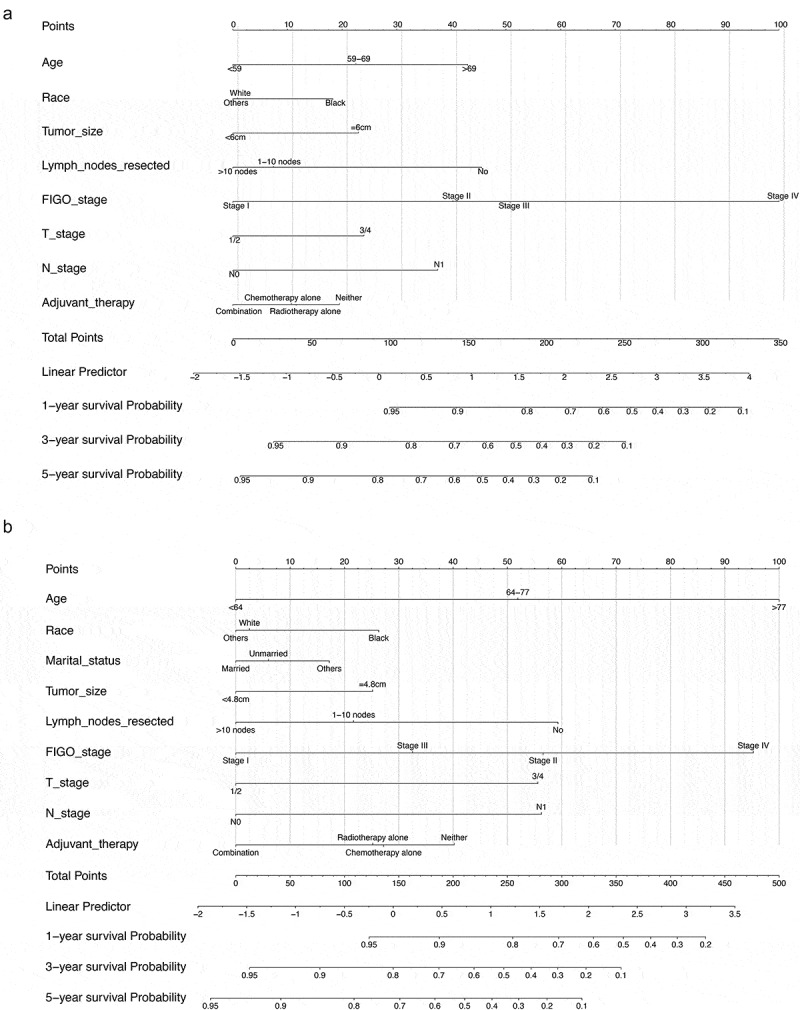
(a): The OS nomogram model. (b): The CSS nomogram model

We constructed nomogram models using the clinical characteristics of age, race, tumor size, number of lymph nodes resected, FIGO stage, T/N stage, and adjuvant therapy to predict OS and CSS based on the multivariate Cox regression analysis results. In the nomograms, the length of the line corresponds to the influence of the different variables, and the different values of the variables correspond to outcomes. The OS nomogram showed that FIGO stage had the maximum impact on prognosis, followed by age, number of lymph nodes resected, and N stage (Figure 4(a)). The CSS nomogram showed that age and the FIGO stage had a considerable influence on CSS, followed by the number of lymph nodes resected, T stage, and the N stage (Figure 4(b)). Each number/category of these variables was assigned a score on a points scale. After the total score was calculated and it was located on the total points scale, a straight line drawn to the 1-, 3-, and 5-year survival probability scale showed the estimated OS or CSS at each time point.

### Clinical evaluation of the nomogram models

**Figure 5.**
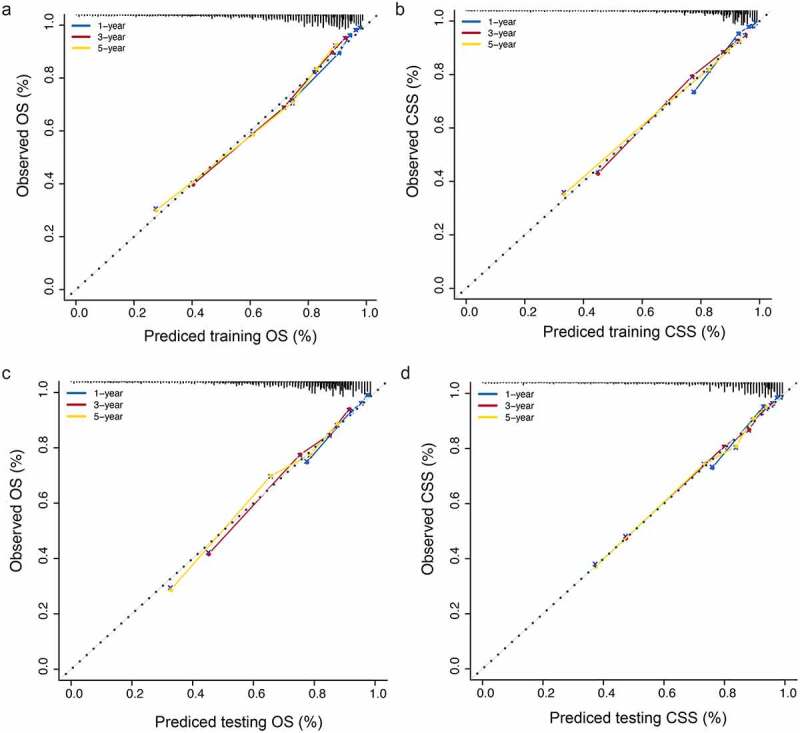
(a): OS calibration diagram in the training set. (b): CSS calibration diagram in the training set. (c): OS calibration diagram in the validation set. (d): CSS calibration diagram in the validation set

To evaluate the accuracy of the nomograms, we drew a calibration curve on each nomogram. The calibration diagrams showed consistency between the predicted values (x-axis) and observed values (y-axis) at 1, 3, and 5 years for OS and CSS, in both the training (Figure 5(a,b)) and validation sets (Figure 5(c,d)), indicating that the model had high accuracy.

### Clinical utility evaluation of the nomograms

**Figure 6.**
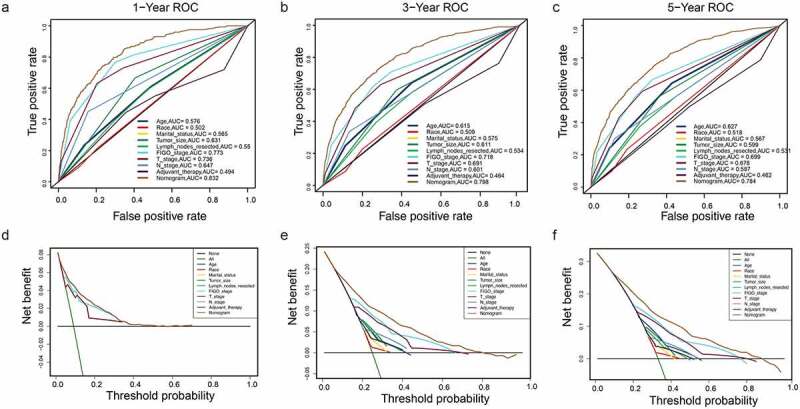
Clinical utility of the nomogram models in the training set. (a–c): ROC curve of each model at 1, 3, and 5 years (OS). (d–f): DCA curve of each model at 1, 3, and 5 years (OS)

**Figure 7.**
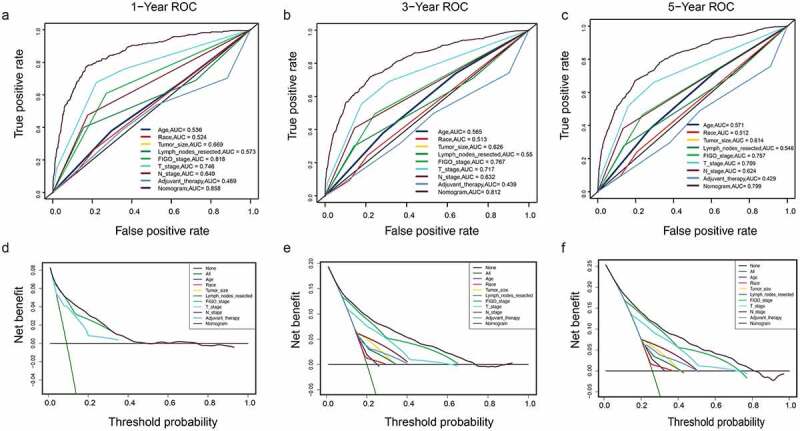
(a–c): ROC curve of each model at 1, 3, and 5 years (CSS). (d–f): DCA curve of each model at 1, 3, and 5 years (CSS)

**Figure 8.**
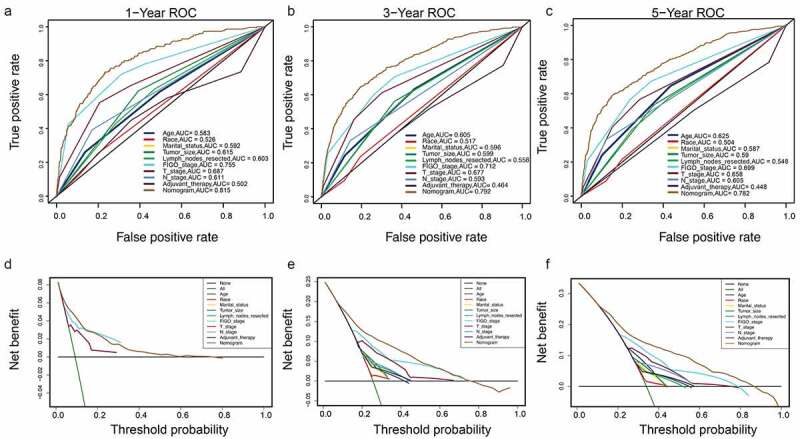
Clinical utility of the nomogram models in the validation set. (a–c): ROC curve of each model at 1, 3, and 5 years (OS). (d–f): DCA curve of each model at 1, 3, and 5 years (OS)

**Figure 9.**
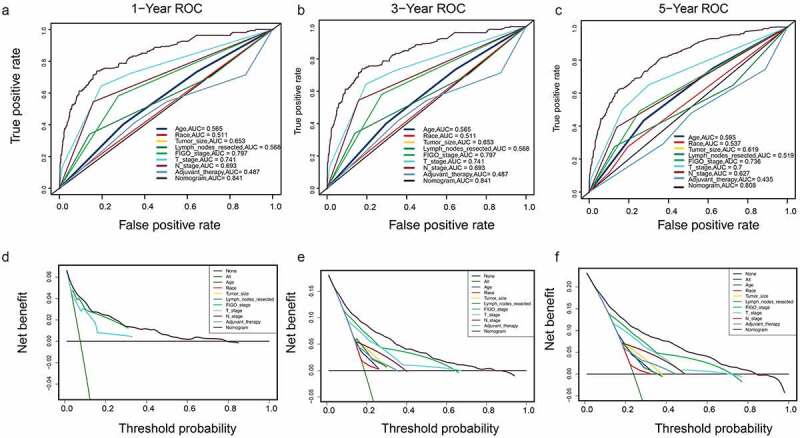
(a–c): ROC curve of each model at 1, 3, and 5 years (CSS). (d–f): DCA curve of each model at 1, 3, and 5 years (CSS)

A DCA is a simple method to evaluate clinical prediction models, diagnostic tests, and molecular markers. To demonstrate the advantages of the nomogram models in the training set, we compared the 1-, 3-, and 5-year ROC curves of single variables (age, FIGO stage, marital status, tumor size, T/N stage, and adjuvant therapy) (Figure 6(a–c)). The results showed that the 1-, 3-, and 5-year area under the curve (AUC) values of the OS nomogram model were the higher 0.832, 0.798, and 0.784, respectively). The 1-, 3-, and 5-year AUC values of the CSS nomogram model were 0.858, 0.812, and 0.799, respectively (Figure 7(a–c)). The net benefits from each nomogram were higher than those from a single clinical variable in the 1-, 3-, and 5-year DCA curves (Figures 6(d–f), 7(d–f)). These results demonstrated the good clinical utility of the nomograms in predicting the 1-, 3-, and 5-year survival of patients with G3 EACs, in turn confirming their positive net benefits and wide practical probability thresholds. Consistent results were obtained from the validation set (Figures 8(a–f), 9a–f).

## Discussion

With the continuous progress of medical diagnosis and treatment, the survival outcome of EC has been significantly improved, and almost 75% of ECs can be diagnosed in the early stages (FIGO stages I or II) [7,8]; however, some patients with ECs still have poor prognoses. The prognoses of patients with ECs are closely related to independent risk factors such as stage, grade, histological type, lymph node metastasis [9]. However, these pathological parameters are still inadequate to predict the rates of survival and recurrence in patients with EC. In recent times, there have been many studies about the accurate prediction of the prognoses of patients with EC. An analysis of the data of 63,729 patients with ECs in the SEER Program database from 1988 to 2015 showed that the CSS nomogram was constructed using age, race, histological grade, clinical stage, tumor size, and the OS nomogram was constructed using histological grade, clinical stage, tumor size, and race, with C-indices of 0.859 and 0.782, respectively [10]. However, the survival outcomes of patients with ECs of different stages, grades, and histological types are considerably different due to their high heterogeneity and different molecular characteristics.

Type I EACs account for about 65% of EACs and are associated with favorable prognoses. Type II mainly includes serous carcinoma, clear cell carcinoma, and carcinosarcoma, which are associated with low incidence, high grade of malignancy, and poor prognosis. The Bokhman dichotomy has certain limitations in predicting the prognoses of patients with ECs. In 2020, the inclusion of The Cancer Genome Atlas (TCGA) molecular typing in the clinical practice guidelines for EC diagnosis and treatment was recommended by the National Comprehensive Cancer Network and European Society of Gynecological Oncology(ESGO)/European Society for Radiotherapy and Oncology (ESTRO)/European Society for Pathology [11]. Although most tumors with high copy numbers and p53 abnormalities are usually serous ECs, quite a few of them are G3 EACs [12,13]. In 2016, the European Society for Medical Oncology-ESGO-ESTRO guidelines defined the high-risk of recurrence group of EC as (I) endometrial carcinoma (type 1), FIGO stage IB, grade 3 tumor (T1/G3 EAC); (II) non-endometrioid carcinoma (type 2); and (III) advanced endometrial cancer, regardless of the pathological type [14]. The prognoses of patients in the high-risk recurrence group are poor, and the risk of recurrence and metastasis is high [15,16]. Therefore, it is important to evaluate the high-risk recurrence of EC to improve the overall survival rate of the patients. At present, there are still some controversies regarding the G3 EAC pathogenesis, prognosis, and treatment.

The C-indices of the training and validation sets in a previous study were 0.814 and 0.837, respectively, and the AUC was 0.7. These were derived by analyzing the prognostic risk factors of 1172 patients with low-grade endometrial carcinosarcoma, indicating that the constructed nomogram had good predictive ability [17]. Another study showed that the mortality of patients with G3 EACs was 45% lower than that of patients with endometrial carcinosarcoma [18]. The 5-year OS of patients with G3 stage I, II, and III ECs were 77.5%, 62.7%, and 49.6%, respectively, indicating poorer prognoses than those of G1 and G2 ECs, while being slightly better than those of endometrial papillary serous carcinoma and clear cell carcinoma [19]. Therefore, some scholars have proposed that G3 EACs should be classified as type II. However, there has been no large-scale retrospective study about G3 EACs.

In this study, we retrospectively analyzed the data of 11,519 patients with G3 EACs registered in the SEER Program database between 2004 and 2015. The univariate Cox regression analysis showed that age, race, tumor size, number of lymph nodes resected, FIGO stage, T/N stage, and adjuvant therapy were independent prognostic factors in terms of either OS or CSS. Previous studies have identified some of these variables as being associated with the survival of patients with ECs [20–22]. These clinical features were used to construct nomograms to predict the prognoses. The accuracy and clinical utility of the nomograms were tested by calibration plots and DCA, respectively. The DCA is a new method to evaluate diagnostic tests and prediction models. Our study had good clinical application value. For OS, the 1-, 3-, and 5-year AUC values of the training set were 0.832, 0.798, and 0.784, respectively. For CSS, the 1-, 3-, and 5-year AUC values were 0.858, 0.812, and 0.799, respectively. This study was the first large-scale retrospective study on G3 EACs. This is the first time that nomograms and websites for patients with G3 ECs based on SEER data have been created. Compared with previous nomograms, our research provides clinicians a more convenient and accurate prediction method. The variables required for our nomograms are common and easy to obtain in clinical practice, making them more cost-effective than other prediction methods that use TCGA molecular typing or biomarkers [23].

However, our study has some limitations. First, the SEER database still lacks some clinical information that is significant for the prognosis, such as invasion of the lymphovascular space. Additionally, there is no molecular profile information, which is likely to be the future trend for precision cancer therapies. Second, the nomograms were based on data retrospectively obtained from the SEER database. A robust nomogram needs to be verified externally in multi-center clinical trials and prospective studies. In the future, we plan to explore the possibility of including more predictors to further improve the performance of the nomograms.

## Conclusions

Our prognostic nomograms will provide a new method to accurately predict the survival of individual patients with G3 EACs.

## Supplementary Material

Supplemental Material

## Data Availability

Some or all data, or code generated or used during the study are available from the corresponding author by request. https://seer.cancer.gov/

## Highlights

1. Marital status affects the survival of patients with G3 EAC.
2. FIGO stage has a great influence on the prognosis of G3 EAC patients.
3. The nomogram has good clinical applicability in predicting the survival of G3 EAC patients
