# Supplementary figures and images for: Construction and validation of nomograms for predicting the prognosis of grade 3 endometrial endometrioid adenocarcinoma cancers: a SEER-based study
Source: Bioengineered. 2021 May 11;12(1):1752–65. doi: 10.1080/21655979.2021.1922247 (PMC8806337; doi:10.1080/21655979.2021.1922247)

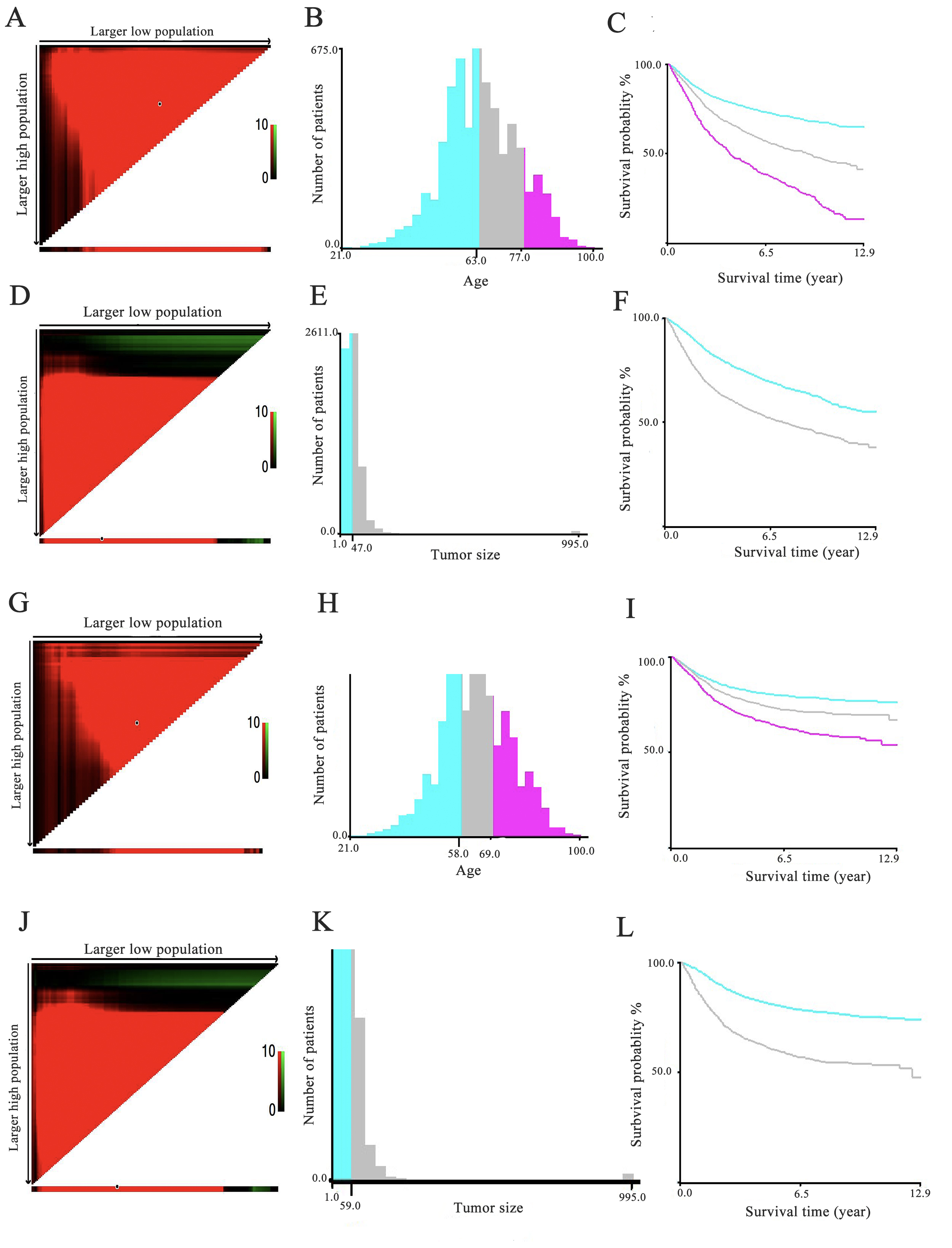

Supplement: Supplemental Material [file KBIE_A_1922247_SM2864.zip › Supplementary Figure1 .jpg]

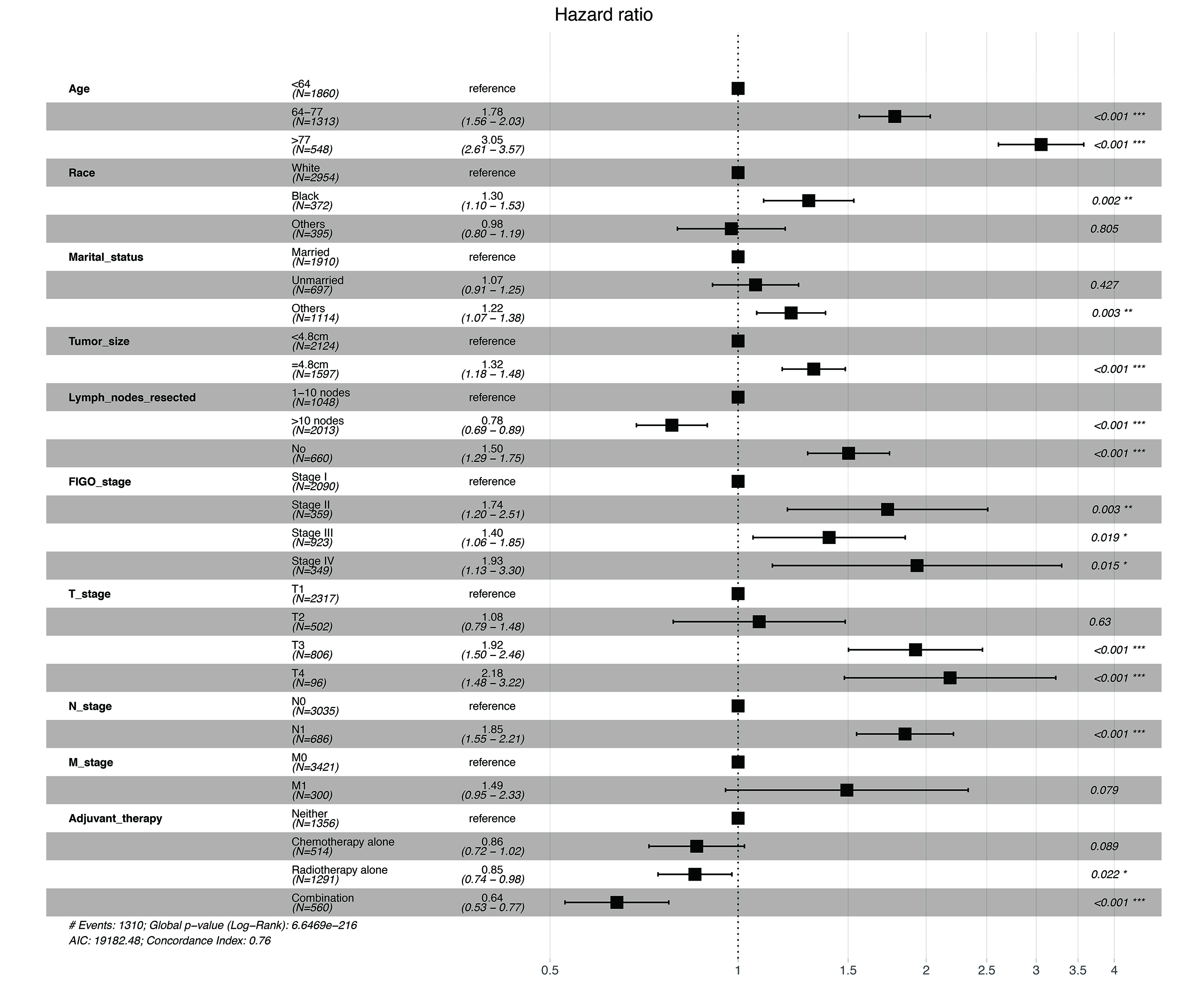

Supplement: Supplemental Material [file KBIE_A_1922247_SM2864.zip › Supplementary Figure2.jpg]

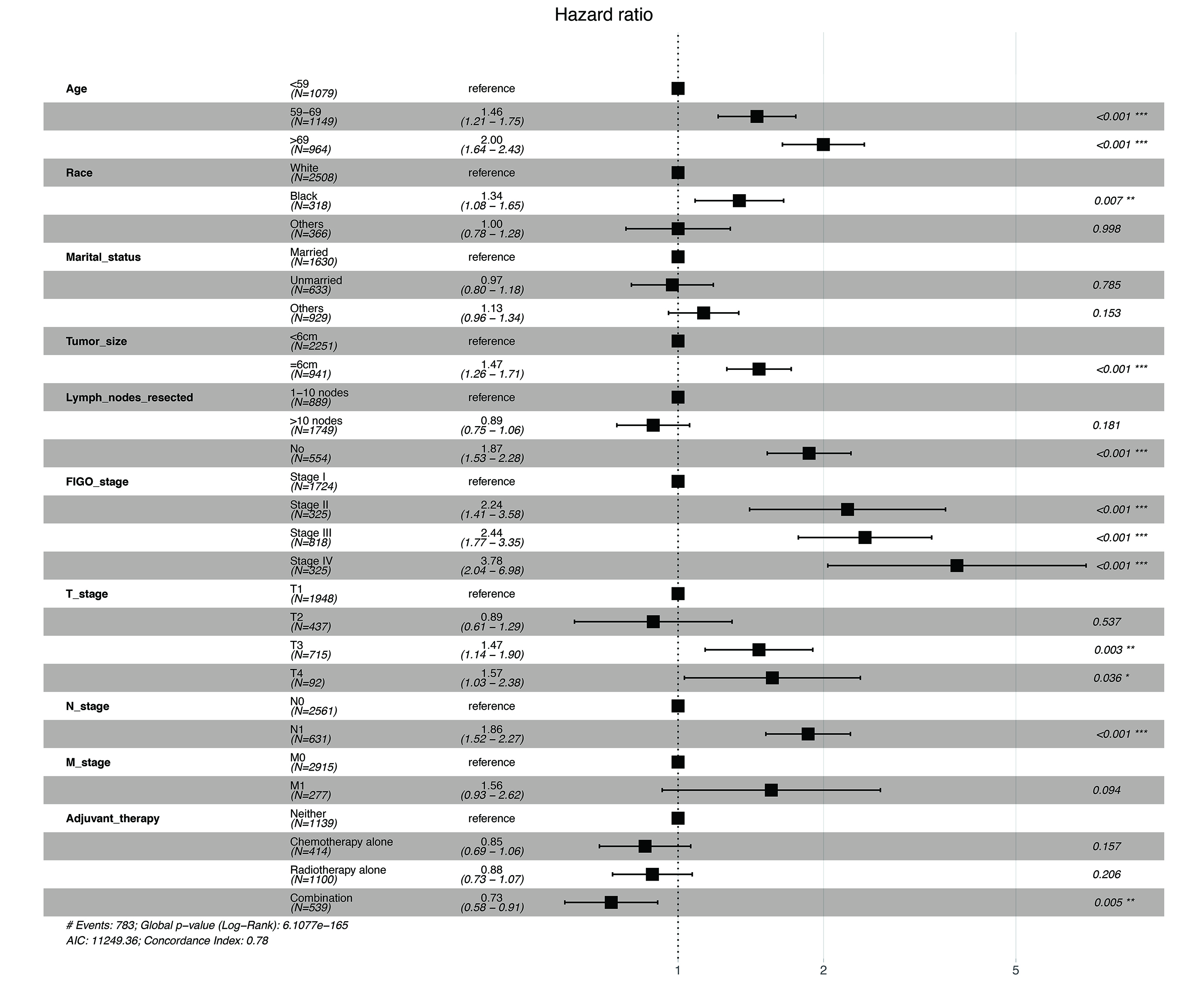

Supplement: Supplemental Material [file KBIE_A_1922247_SM2864.zip › Supplementary Figure3.jpg]
